# A Feasibility and Acceptability Trial of Social Cognitive Therapy in Early Psychosis Delivered Through a Virtual World: The VEEP Study

**DOI:** 10.3389/fpsyt.2020.00219

**Published:** 2020-03-25

**Authors:** Andrew Thompson, Farah Elahi, Alba Realpe, Max Birchwood, David Taylor, Ivo Vlaev, Fiona Leahy, Sandra Bucci

**Affiliations:** ^1^Orygen, the Centre for Excellence in Youth Mental Health, Melbourne, VIC, Australia; ^2^Warwick Medical School, University of Warwick, Coventry, United Kingdom; ^3^Population Health Sciences, Bristol Medical School, University of Bristol, Bristol, United Kingdom; ^4^Department of Surgery & Cancer, Faculty of Medicine, Imperial College London, University of London, London, United Kingdom; ^5^Behavioural Science Group, Warwick Business School, University of Warwick, Coventry, United Kingdom; ^6^Division of Psychology and Mental Health, Faculty of Biology, Medicine and Health, School of Health Sciences, Manchester Academic Health Sciences, Manchester, United Kingdom; ^7^Greater Manchester Mental Health NHS Foundation Trust, Manchester, United Kingdom

**Keywords:** social cognition therapy, virtual environments, virtual world, first episode psychosis, psychosis, proof-of-concept trial

## Abstract

**Background:**

Addressing specific social cognitive difficulties is an important target in early psychosis and may help address poor functional outcomes. However, structured interventions using standard therapy settings including groups suffer from difficulties in recruitment and retention.

**Aims:**

To address these issues, we aimed to modify an existing group social cognitive intervention entitled ‘Social Cognition and Interaction Training' (SCIT) to be delivered through a virtual world environment (Second Life ©).

**Methods:**

A single arm nonrandomized proof-of-concept trial of SCIT-VR was conducted. Five groups of three to five individuals per group were recruited over 6 months. Eight sessions of SCIT-VR therapy were delivered through the virtual world platform Second Life© over a 5-week intervention window. Feasibility was examined using recruitment rates and retention. Acceptability was examined using qualitative methods. Secondary outcomes including social cognitive indices, functioning, and anxiety were measured pre- and postintervention.

**Results:**

The SCIT-VR therapy delivered was feasible (36% consent rate and 73.3% intervention completion rate), acceptable (high overall postsession satisfaction scores) and safe (no serious adverse events), and had high levels of participant satisfaction. Users found the environment immersive. Prepost changes were found in emotion recognition scores and levels of anxiety. There were no signs of clinical deterioration on any of the secondary measures.

**Conclusion:**

This proof-of-concept pilot trial suggested that delivering SCIT-VR through a virtual world is feasible and acceptable. There were some changes in prepost outcome measures that suggest the intervention has face validity. There is sufficient evidence to support a larger powered randomized controlled trial.

**Clinical Trial Registration:**

ISRCTN, identifier 41443166

## Introduction

Novel treatments targeted at functional outcome in early psychosis are important as functional recovery is less common than symptomatic recovery (1), and the two are not always intrinsically linked (2). Continued functional impairment has significant impact both personally and economically and is often rated as the most important treatment goal in people experiencing their first episode of psychosis (3). One treatment approach targeting functional outcomes broadly is social cognition remediation therapy. Social cognition, including the domains of emotion recognition, theory of mind, and attributional bias, is often poor in both established psychosis (4) and first episode psychosis (5), is strongly related to social functioning (6), and contributes unique variance in predicting functional outcome (7, 8). Although varied in approach, social cognitive remediation programs all appear to improve social cognition measures (9). Global approaches that deliver both social cognition skills training and real-world application of these skills also appear to improve functional outcome at least in established psychosis (9). In a previous study, we adapted one of these established global type social cognition programs, Social Cognition and Interaction Training (10), in an Australian sample of first episode psychosis patients (11). We found in a small pilot study that this group intervention was acceptable to young people, but feasibility was low as consent rate and maintaining attendance in the group was relatively poor (11). For some young people, the thought of attending traditional individual or group therapy provoked considerable anxiety (11). In this sense, we were aware that our intervention was not reaching a wider group who might benefit from social cognition training, especially as poor social cognition is associated with anxiety and negative symptoms (12).

Over the last few decades, technological approaches to delivering healthcare have developed rapidly. This is also the case in mental health problems where the use of apps and social media sites has been used to encourage engagement and widen the reach of interventions to other populations (13). Although emerging, the use of technology to deliver interventions has received relatively little attention in the psychosis field, perhaps due to concerns regarding young people with psychosis not using technology or that technology may be unhelpfully incorporated into their symptoms (14). However, there is no consistent evidence to suggest either of these concerns are the case (13, 15).

Virtual Reality (VR) interventions are showing promise in engaging and helping young people with psychosis often by providing safe exposure to challenging environments. Virtual world platforms are being used to deliver physical and psychological treatments to people with a range of health problems, including obesity, autism, intellectual disabilities, and diabetes (16–19). A virtual world is an online shared community environment where members can interact in a custom-built, simulated world. The community interacts in the simulated world using either text-based, 2-D and/or 3-D graphical models called avatars (20). Although under-researched, virtual world platforms have the potential to reduce communication barriers and improve access to support and treatment in people with mental health disorders (21).

One of most widely used virtual world environments is the online and freely available social world Second Life©. Second Life© has been used as a virtual world platform in healthcare education, delivery, and engagement and has considerable potential in delivering psychological therapies (21, 22). For example, Second Life© has been used to treat social anxiety and has been found to be feasible and acceptable with effects sizes potentially comparable to face-to-face Cognitive Behavior Therapy (CBT) for Seasonal Affective Disorder (23) and in treating social cognition problems in autism (24). However, we know of no other study that has used Second Life© in the treatment of psychosis. We adapted a traditionally face-to-face delivered social cognition and interactional training (SCIT) (25, 26) to a virtual world in Second Life© (SCIT-VR) using a codesign process involving young people, clinicians and web designers. This work is described in detail elsewhere (27). The title of this trial is “virtual reality as a method of delivering social cognitive therapy in early psychosis (VEEP).” Our aim was to test the feasibility and acceptability of the SCIT-VR intervention approach in a group of young people who were recovering from a first episode of psychosis.

## Methods

### Study Design

Single arm, nonrandomized, proof-of-concept study.

#### Development of SCIT-VR

**Figure 1:**
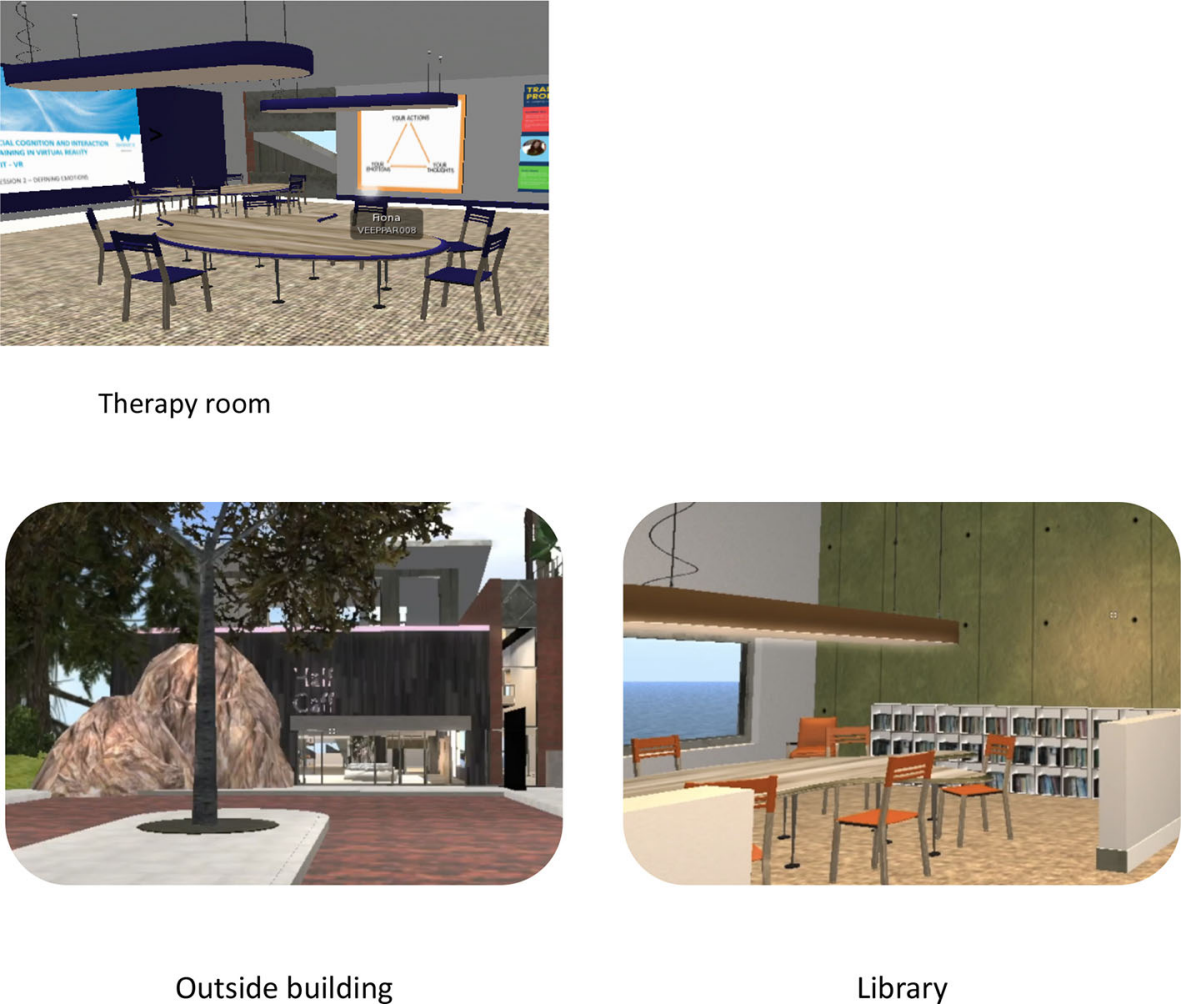
Screenshots of the Second Life© environment adapted from (20).

We undertook a modified codesign process with 20 young service users in total, two carers, a virtual world designer, and the study team. The co-design process is described in detail elsewhere [(27); **Figure 1** displays screenshots of the environment].

#### SCIT-VR Structure

**Figure 2:**
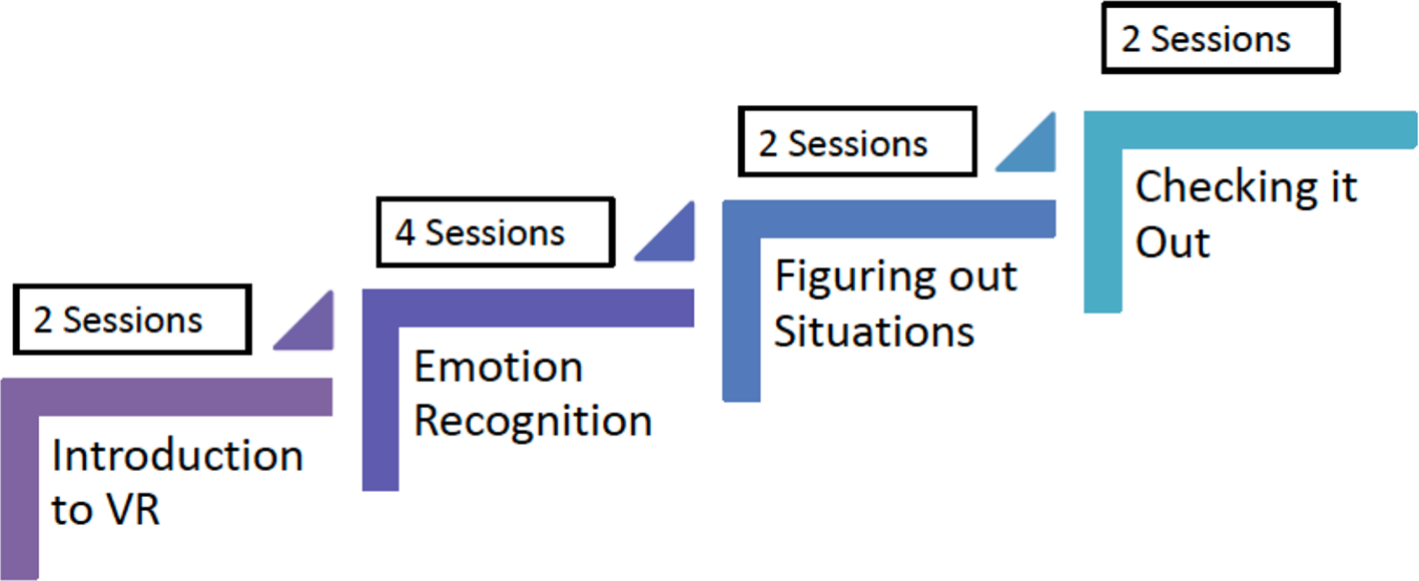
SCIT-VR therapy structure.

SCIT-VR consisted of 10 sessions (two individual and eight group sessions). The original SCIT (25) was delivered in 20 sessions but our adapted face-to-face version in early psychosis with young people living in Australia was delivered in 10 sessions (11). In the SCIT-VR version described in the current study, the group experience sessions at the end of the intervention were truncated based on our codesign feedback. We included two initial 30 minute sessions where the researcher helped the participant to set up and familiarize themselves with the technology in order to take part in the virtual group sessions. This included providing a headset, helping the participant through the login procedure, setting up their avatar, and helping to familiarize themselves with the Second Life© environment. The SCIT-VR content was then delivered over eight one-hour sessions. The basic structure of the intervention was the same as the original SCIT (25). The first three sessions focused primarily on emotion recognition, the next three sessions focused on attribution bias and paranoia as an emotion, and the last two sessions on “skills acquisition” using a CBT framework to discuss examples of social difficulties faced by the participant (see **Figure 2**). The intervention was manualized and delivered by a lead therapist and a cotherapist to groups of five participants.

#### Recruitment and Eligibility Criteria

Participants were recruited primarily from one National Health Service (NHS) early intervention in psychosis services in Coventry and Warwickshire, UK. These services provide a three year period of care to around 120 individuals experiencing their first episode of psychosis. Psychosis-specific interventions include antipsychotic medication, CBT, family interventions, and vocational interventions, plus individual case management. All young people aged between 18 and 45 years registered with the early intervention service and not in an acute phase of psychosis (*i.e.* not in hospital/in contact with the crisis team) were eligible. Exclusion criteria included a confirmed diagnosis of a learning disability or a neurological disorder.

### Measures

#### Feasibility and Acceptability

Feasibility was assessed using consent rates in those eligible, levels of session completion and follow-up rates (28). Study completers were classified as individuals who attended at least six of the eight sessions (the two set-up sessions and at least four sessions). Acceptability was assessed immediately following each session by asking participants to complete a five-item feedback questionnaire completed online through the dedicated study website. Acceptability was also assessed using a semi-structured interview with participants post-intervention. We also completed a focus group with clinicians whose patients were involved in the study to access clinicians' views to real world applicability. These findings will be discussed in a future paper. Interview data was thematically organized.

#### Outcome Measures

##### Social Cognition

1) Social Cognition Screening Questionnaire (SCSQ)—comprises of 10 short vignettes, which presents an interaction between a fictional character and the participant. It has five subscales: verbal memory, schematic inference, theory of mind, metacognition, and hostility bias (29). 2) Bell Lysaker Emotion Recognition Task (BLERT)—this is a test of one's ability to correctly identify seven emotional states using facial emotion recognition (and body cues) endorsed by the preliminary reports of the SCOPE study (30). 3) Hinting task (31)—a theory of mind task where participants are required to identify verbal hints in a conversation. The social cognition measures were informed from the results of the ongoing Social Cognition Psychometric Evaluation (SCOPE) study (30, 32). 4) Cognitive Style Questionnaire-Short Form (CSQ-SF; (33)—focuses on 24 hypothetical positive and negative events relating to successes and failures in academic achievement, employment, and interpersonal relationships. This questionnaire assesses how an individual attributes the outcome of events.

##### Social Function

Personal and Social Performance Scale [PSP; (34, 35)]—clinician reported measure to assess the severity of social functioning in those with acute symptoms of schizophrenia.

##### Quality of Life

EuroQual 5-D (36) scale—a widely used scale assessing quality of life through five items and a visual analog health “thermometer” rating.

##### Behavioral Change Intention

1) Theoretical Domains Framework—Domain Four (TDF—D4)—used to develop questionnaires including 12 theoretical domains of potential behavioral determinants. Domain four is called *Beliefs about capabilities* and measures participants' self-efficacy, self-confidence, and perceived competence (37).

2) Theoretical Domains Framework—Domains Eight and Nine—these correspond to *Intentions* and *Goals* in the Framework. *Intentions* involves measuring participants' decision to undertake a behavior or resolution in a particular way. *Goals* involves assessing participants' goal priorities, targets, and their intention to implement such goals into their life (37).

##### Presence in the Virtual World

Presence (a VR concept employed to test the extent to which the participant was “immersed” in the virtual world) was tested using the presence questionnaire (38).

##### Psychopathology

Brief Psychiatric Rating Scale (BPRS) (39)—assesses participants' psychopathology and symptom severity. Researchers were trained on administering this scale to gold standard ratings.

##### Neurocognition

1) National Adult Reading Test (NART)—a commonly used method in clinical settings for estimating premorbid intelligence levels of English-speaking individuals (40). 2) Trail Making Test—a widely used neuropsychological test of visual attention and task switching (41).

##### Adverse Events

These were collected in line with the University of Warwick Clinical Trials operating procedure.

### Procedure

Eligible individuals were identified by the treating team. Potential participants were approached for consent by the research assistant. Consented participants completed the baseline assessment over a maximum two weeks. When at least three but preferably five participants were consented and baseline assessment completed, therapy sessions commenced. The time between recruitment of the first and last participant was short (no longer than six weeks). The two initial individual sessions were arranged before the group sessions were scheduled at the convenience of the participants. To encourage attendance, participants were contacted by text prior to each session. Participants were aware that the secondary therapist was available *via* a study phone for an hour prior and after each session both for safety reasons and to help with technology queries.

The intervention was delivered to groups of three to five participants. The primary and secondary group facilitators were psychology trainees educated at masters level/above. The intervention was manualized. A number of PowerPoint presentations and videos were retained from the original SCIT intervention and delivered through Second Life© as part of the sessions. Immediately following each session, participants were taken to a ‘virtual library' where they were invited to complete a session feedback sheet. Feedback was not available to the therapists until after the end of the intervention period. At the end of the 10 therapy sessions, participants completed the post-intervention assessments. They were also invited to complete a 30-minute interview with a research assistant to discuss views on the technology, the virtual world and the intervention itself. All interviews were topic-guide driven, audio-recorded and transcribed. Ethical approval for the study was obtained from the West Midlands—Solihull Research Ethics Committee.

### Data Analysis

For the secondary outcome measures, pre- and post-test scores were compared using basic t tests. Demographic information on the participants was also collected and presented. Qualitative interviews were conducted post intervention, using a semi structured schedule. Interviews were conducted until data saturation was achieved (N = 15). A thematic analysis (42) method was used to analyze the data. Analyses were conducted using the software NVivo.

## Results

### Sample Characteristics and Feasibility

Descriptive data for the participants who consented to take part in the study (*N* = 19) are displayed in **Table 1**. More males than females were recruited and the age was relatively young (mean 25.6 years). IQ estimates were slightly above normal and the level of psychopathology was relatively high. Of all the young adults with First Episode Psychosis (FEP) in the service who were deemed eligible by the care coordinators and approached (53 over a 6-month period), 19 participants (36%) agreed to take part. Four of those participants who consented (21%) then withdrew from the study before completing outcome measures, leaving outcome data for 15 individuals. Reasons for withdrawal from the study were: unable to commit the time (n = 2); experiencing a recent bereavement (n = 1); not able to contact (n = 1). If participants attended four or more of the total eight sessions, they were considered a completer. Eleven of the 15 participants were deemed completers (73.3%). The mean (SD) number of VEEP sessions attended by participants was 4.94 (2.64), which was a mean attendance rate of 61.8% of the total eight available sessions.

**Table 1:** Participant demographics, psychopathology and neurocognitive measures at baseline.

Variable	Value	
**Age**	Mean	25.61
	SD	6.49
**Gender**	Male	14
	Female	5
**Highest level of education**	A level	3
	Trade or technical training (incomplete)	5
	Trade or technical training (complete)	6
	Tertiary diploma/certificate	2
	Undergraduate degree (incomplete)	1
	Undergraduate degree (complete)	2
**Psychopathology (BPRS rating at baseline)**	Mean	38.21
	SD	6.35
**NART score (premorbid intelligence)**	Mean	28.06
	SD	7.78
**Trail making task (visual attention and task switching)**	Trail A mean time (secs)	35.95
	SD	11.63
	Trail B mean time (secs)	113.58
	SD	74.28

### Acceptability

#### Feedback Questionnaires

Participants gave ratings out of five for each of the five items assessing acceptability on a feedback questionnaire completed after every session. Acceptability was rated >3 out of five on every item. Descriptive data for ratings of each item of the questionnaire are presented in **Table 2**, while total ratings (0–25) for each intervention session are presented in **Table 3**.

**Table 2:** Acceptability ratings (0–5) for each item of the feedback questionnaire.

**Questionnaire item**	**Mean**	**SD**	**Min**	**Max**
Q1:Suitable level of content (*e.g.* easy to understand)	4.40	0.54	3	5
Q2:Relevance and value of the content	4.16	0.56	3	5
Q3:Guidance from the therapist	4.69	0.51	4	5
Q4:Encouragement to participate and interact	4.57	0.67	3	5
Q5:Safety of the VR world	4.70	0.61	2	5

**Table 3:** Total acceptability ratings (0–25) for each session of VEEP.

**Session**	**Mean**	**SD**	**Min**	**Max**
1: SCIT-VR and social cognition definition	22.11	1.62	19	25
2: Defining emotions	21.71	2.06	19	24
3: Guessing people's emotions	22.33	1.94	19	25
4: Suspicious feelings	22.25	2.63	20	25
5: Jumping to conclusions	22.6	2.88	19	25
6: Separate facts from guesses & gathering more information	23.75	1.26	22	25
7: Checking it out—part I	22.6	2.3	19	25
8: Checking it out—part II	24	0	24	24

Participants were also given the opportunity to provide a written feedback response on the questionnaire. Written responses are presented in **Supplementary Table 1**.

### Qualitative Interviews

Categories and illustrative quotes from the individual interviews are represented in **Table 4**. These were related to investigator's concerns on safety and immersion/realism in the environment. Participants did not have specific concerns regarding safety, liked the anonymity, and found the environment relatively immersive. There were some issues with the technology reported by the participants.

**Table 4:** Feedback and illustrative quotes.

**Nature of the Feedback**	**Illustrative Quotation**
*Feedback on the virtual world*
Privacy and Safety	"I think it was safe yeah no I don't think erm I had any concerns of like you know people listening in who shouldn't be or people able to access...” (Participant 017)
Anonymity	“Cause I didn't know them, I didn't think it would be too much of an issue. So I said some certain things about like my psychosis.” (Participant 005)
Second Life Environments	“I thought that was pretty good with the different rooms, the different therapy rooms.” (Participant 015)
Sense of Realism	“you know the graphics could've been a bit better? “ “it may-may have made me feel like I was in more of a real life setting and which would've been oh-okay because it would've got me out of my comfort zone.” (Participant 005)
*Feedback on the treatment*	
Treatment content and delivery	“I think the content was delivered...comprehensively during the presentations, during the sessions, I didn't feel I needed to go back and re-read anything.” (Participant 017)
Impact of treatment on wellbeing	“About...you know...what people, what you think they might mean and what they actually mean. You know like with facial expressions, you-you can come up and say, ‘Oh were they were they giving me a dirty look?' When actually if you think about it they may be having a bad day or there maybe other reasons so...yeah I found that useful.” (Participant 007)
Support during the treatment	“Not much. Literally not much. Everything (Research Associate's name) set up perfectly. Like she put it on and all I had to do ‘cause she even saved my login details so I didn't even have to put them in once.” (Participant 005)
*Ideas for improvement*	
Technological Difficulties	"I would log in five minutes before the session, my computer would crash, and I would spend the next ten minutes trying to login, and I'll be five minutes late" (Participant 016)
Duration and timing of treatment	“Cause the times they were aren't ermm like you said yeah I think evenings maybe better for some people. Depending on you know, I suppose age and whether they work or not. “ (Participant 007)

### Secondary Outcome Measures

**Figure 3:**
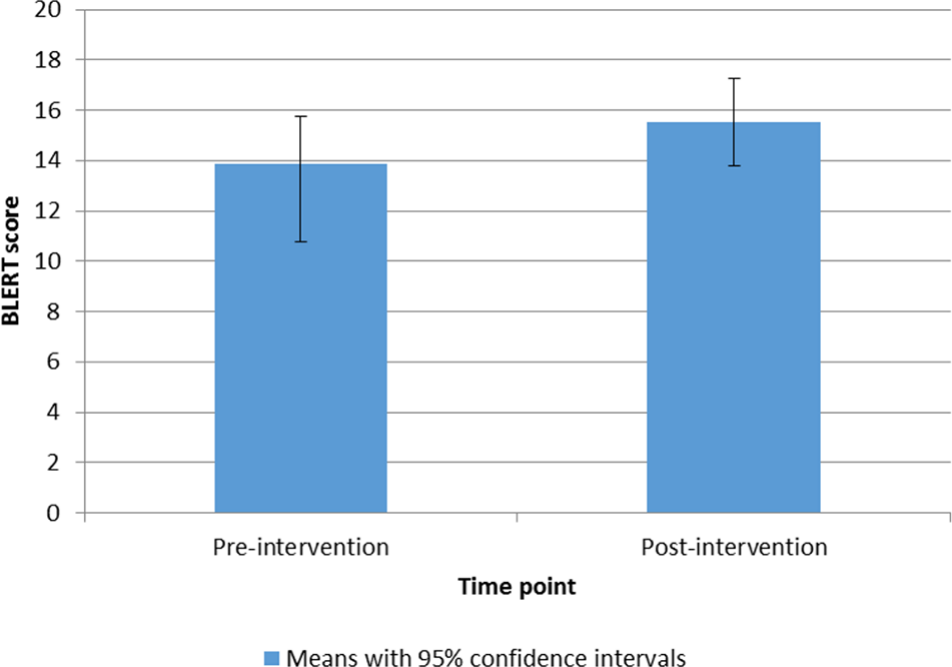
Bar chart to show means and 95% confidence intervals of BLERT scores from pre- to post-intervention.

**Figure 4:**
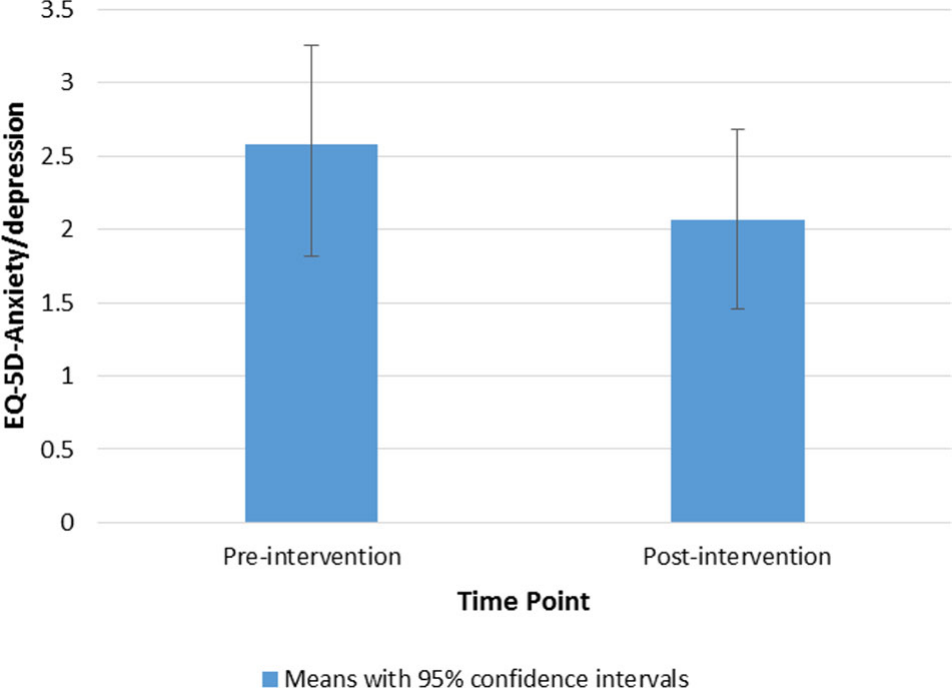
Bar chart to show means and 95% confidence intervals of EuroQual-5D-Anxiety/depression subscale ratings from pre- to post-intervention.

Treatment outcome was defined as change in social cognition, social function, quality of life, and behavioral change intention. Paired t-tests showed that there was a significant increase in emotion recognition (BLERT) scores from pre- to post-intervention, with a medium effect size (Hedges' g), t(14) = 3.21, p = .006, *g* = 0.58, (see **Figure 3**). There was also a significant decrease in the anxiety/depression subscale of the EuroQual-5D, indicating an improvement with a medium effect size, t(14) = 2.43, p = .029, *g* = 0.41 (see **Figure 4**) (43, 44). There were no other significant differences from pre- to post-treatment on any of the other measures (p > .1). See **Table 5** for pre- and post-intervention analyses. Presence was measured in the last nine consecutive participants (due to an omission in the interview schedule). The results showed that the participants had a reasonable degree of presence in the virtual world (average score on the presence questionnaire (38) 154.2, SD 18.8 see **Supplementary Figure 1**).

**Table 5:** Pre- and post-intervention means and SDs for secondary outcomes.

Variable		Pre-intervention		Post-intervention		p value	Hedges' g
		Mean	SD	Mean	SD		
BPRS	Total	38.21	6.35	36.4	9.16	0.348	0.23
	Anxiety	2.16	1.26	2.33	1.35	0.843	0.13
	Depression	2	0.94	2.13	1.19	0.818	0.12
SCSQ	Total SCSQ	31.13	3.52	33.29	3.4	0.253	0.61
	Theory of mind	7.05	2.2	8	1.56	0.173	0.49
	Schematic Inference	6.95	1.27	7.67	1.23	0.126	0.56
	Verbal memory	7.89	1.15	8.33	1.23	0.164	0.36
	Metacognition	9.24	0.77	9.29	0.64	0.823	0.07
	Hostility bias	1.68	1.25	1.47	1.36	0.855	0.16
BLERT		13.84	4.36	15.53	3.16	0.006	0.58
Hinting task		17.84	2.39	18.67	1.72	0.344	0.39
CSQ-SF	CSQ-SF-Total	195.16	27.4	191.53	24.2	0.13	0.14
	Internality subscale	47.32	5.7	49.53	4.87	0.337	0.41
	Globality	43.21	8.16	43.2	7.1	0.453	0
	Stability	42.58	6.55	39.67	6.95	0.098	0.42
	Negative Consequences	21.32	4.73	20.13	4.63	0.219	0.25
	Self-worth implications	40.47	9.32	38.67	8.93	0.067	0.19
PSP		66.74	15.65	67.87	14.03	0.773	0.07
EuroQual-5D	Total	8.74	3.81	8.6	3.79	0.187	0.04
	Anxiety/depression	2.58	1.3	2.07	1.1	0.029	0.41
EuroQual-VAS		71	16.92	70	21.04	0.669	0.05
TDF-4 (Beliefs about capabilities)	Total	2.57	0.55	2.38	0.56	0.486	0.33
	Group	2.64	0.73	2.55	0.75	0.673	0.12
	Individual	2.51	0.48	2.33	0.57	0.331	0.33
TDF-8 (intentions)	Total	2.48	0.82	2.21	0.61	0.576	0.37
	Group	2.56	0.8	2.24	0.68	0.454	0.45
	Individual	2.4	0.88	2.18	0.63	0.777	0.28
TDF-9 (Goals)	Total	2.98	0.73	2.72	0.56	0.399	0.39
	Group	3.03	0.7	2.68	0.68	0.274	0.49
	Individual	2.93	0.81	2.76	0.52	0.772	0.25

The pre- and post-intervention means (SD), t values, p values, and effect sizes (Hedges's g) for level of psychopathology (total Brief Psychiatric Rating Scale—BPRS), anxiety and depression subscales of the BPRS, total and each subscale of the Social Cognitive Screening Questionnaire (SCSQ), Bell Lysaker Emotion Recognition Task (BLERT), hinting task, total and each subscale of the Cognitive Screening Questionnaire-Short Form (CSQ-SF), Personal and Social Performance scale (PSP), Euro-Qual-5D Quality of life Scale (EuroQual-5D) -total and anxiety/depression subscale, EuroQual-Visual Analogue Scale (EuroQual-VAS), Theoretical Domains Framework (TDF) 4, 8, & 9 totals, group and individual ratings are presented.

## Discussion

We conducted a proof-of-concept nonrandomized study of an established group social cognition intervention (SCIT) adapted for delivery through a virtual world (Second Life©), SCIT-VR. We found that the intervention was generally feasible as the intervention completion rate was reasonable, and the delivery of the intervention by relatively inexperienced clinicians in an NHS setting was possible. Our completion rate is similar to that of the trial conducted by Bartholomeusz et al. (11) where 75% of participants completed the intervention. The intervention was mostly considered acceptable as measured by attendance rate, post-session feedback questionnaires and qualitative interviews. There were no concerns about safety either from adverse events or from individual participant interviews. There was a suggestion that the intervention had face validity as pre-post changes were observed on measures of social cognition and anxiety. There was no change in participants' intention to change or self-efficacy scores. The intervention was not primarily aimed at improving intention to engage in further therapy or social activities but we anticipated this might be a beneficial effect of the approach. Further studies might want to consider the use of virtual worlds as interventions aimed more directly at engagement and self-efficacy as part of a pathway to accessing more structured and face-to-face interventions.

The small scale pilot face-to-face version of this study was conducted by Bartholomeusz et al. (11). The average effect size score in their trial was d = 0.29, compared to our g = 0.22. Similarly this trial also found significant improvements on emotion recognition. This is not completely unexpected as we believe the virtual world is a mechanism to provide the SCIT intervention to those who might find it difficult to attend face-to-face therapy. The findings in the current study across a number of measures indicate that the virtual world intervention we have developed has the potential to improve social cognitive domains in those people with early psychosis. However, this is a small pilot study with a small sample size. Therefore, a large randomized controlled trial is required to determine whether providing SCIT in a virtual world can lead to significant improvements in this population.

This is the first study to our knowledge using a virtual world to deliver a structured group therapy in early psychosis. It was relatively ambitious, as a number of social cognition skills would appear to rely on face-to-face social contact to achieve mastery. However, there have been previous studies that have used Second Life© successfully with the aim of improving social cognition skills in those with high functioning autism (24). We believe that the didactic teaching approach that was delivered in Second Life© was possibly more successful than more group-focused sessions as measured by higher levels of feedback on the individual sessions that were more didactic in nature. The sessions with more specific teaching content were rated more highly by the participants than the sessions that involved group work. No participants highlighted that they enjoyed the group or specifically felt part of the group. Future studies using this approach may want to consider whether the intervention needs to be delivered in a group setting. Others, for example, have successfully used an individual approach when using virtual worlds to treat social anxiety (23).

Participants reported a high degree of presence in the virtual environment, which was surprising as the interaction with Second Life© is purely through a computer monitor and headset and not using a head mounted display or more traditional immersive technology and the interview themes also highlighted that people felt high levels of presence in the virtual world. However, the presence questionnaire was completed by a minority of participants. Previous research has attempted to assess the sense of presence using Second Life©; for example, nursing students reported relatively high degree of presence in Second Life©, which was related to attitude towards using the environment (45). We have considered whether a more immersive environment such as traditional VR or using 360 videos might lead to a greater acquisition of social cognition skills as they more approximate real-life situations.

These findings support the growing number of VR interventions for people who experience psychosis. According to a recent literature review, there are a distinct lack of studies examining the potential impact of VR interventions for early psychosis (46) Therefore, our trial is timely. The key advantage of VR is that real-time behavior can be observed when interacting in the VR world. Furthermore, the environment can be modified and controlled to elicit certain responses (47).

There are a number of limitations of the study. Firstly, there was no control group and therefore we cannot be sure that any improvements are due to the intervention. There were challenges in recruiting patients, which impacted on the feasibility of the study, with only 36% of eligible patients consenting to take part in the intervention. This differs from previous research, which showed that two thirds of patients with psychosis were willing to be contacted about taking part in research (48). However consent rates for those eligible participants diagnosed with psychosis differ between trials. For example, while Wood et al. (49) reported a 67% consent rate (30/45), Kanniston et al. (50) reported a 33% consent rate (1139/3417), and Schrank et al. (51) reported a mean consent rate of 40.0%. One possible reason for our recruitment rate is that some potential participants may not have had access to the internet or may have had limited digital skills, reflecting an ongoing concern around digital exclusion for some people. This issue is a concern more generally in digital mental health studies and requires further consideration in finding ways to reduce digital inequalities (52). More frequent contact between clinicians and researchers has been shown to improve recruitment. Therefore this should be reviewed in future research (53).

Whilst we had a reasonable retention rate, we had problems keeping people engaged in the study. There were some problems with the technology during some of the sessions which required the secondary therapist to rectify; on two occasions this was not possible. This was highlighted in the qualitative interviews. One of the limitations of using Second Life © was that it was not functional on tablets or hand-held devices and it could not be streamed through a gaming console. A number of participants did not own a laptop or desktop computer. We were able to loan a computer to participants who did not have access to one, but for those who do not own or have access to a computer participation in an intervention delivered on a platform such as Second Life© is compromised.

Given the results of this small proof-of-concept study, there is sufficient evidence to consider a full-scale efficacy trial in this population. Challenges in designing such a trial are whether to consider adjunctive experiences that might be more immersive such as combining other VR approaches. Based on the feedback, we would also need to consider whether all parts of the intervention need to be delivered in a group setting and how we might be able to overcome technical problems and access to those without a computer. The delivery of evidence based psychological therapies to this group continues to prove challenging, and the use of technologies such as Second Life© shows promise in widening access and engagement for this hard to reach group.

## Data Availability Statement

The datasets generated for this study are available on request to the corresponding author.

## Ethics Statement

This study was carried out in accordance with the recommendations of the 'West Midlands – Solihull Research Ethics Committee' with written informed consent from all subjects. All subjects gave written informed consent in accordance with the Declaration of Helsinki. The protocol was approved by the 'West Midlands – Solihull Research Ethics Committee'.

## Author Contributions

AT, SB, MB, IV, and DT wrote the grant application for this feasibility and pilot trial. AT, AR, DT, and FE were responsible for coordinating the design of the virtual world. AT, AR, and FE conducted the co-design workshops. AT, FE, FL, and AR undertook the trial. AT drafted the manuscript. All investigators have been involved in revising the report, and all authors have seen and approved the final version.

## Funding

The VEEP study was sponsored by the University of Warwick and supported by the PsyIMPACT programme of MQ: MQ16PI100011. Transforming mental health (Register charity in England/Wales: 113991 & Scotland: SC046075).

## Conflict of Interest

SB is a director of a not-for-profit company aimed at commercialising mental health apps.

The remaining authors declare that the research was conducted in the absence of any commercial or financial relationships that could be construed as a potential conflict of interest.
