# Supplementary material for: A Feasibility and Acceptability Trial of Social Cognitive Therapy in Early Psychosis Delivered Through a Virtual World: The VEEP Study
Source: Front Psychiatry. 2020 Mar 25;11:219. doi: 10.3389/fpsyt.2020.00219 (PMC7109496; doi:10.3389/fpsyt.2020.00219)

**Supplementary Table 1:** Feedback responses on questionnaire by session number

| Session 1 | It is good |
| --- | --- |
|  | Really found the video clip interesting |
|  | Amazing session |
|  | It was good to learn about new things |
|  | Good productive session |
|  | Good session |
|  | Very interesting content |
|  | No comment |
|  | Farah explained everything very well and clearly so I could understand what the course is about and what we be doing during the sessions. |
| Session 2 | It felt safe |
|  | Productive session |
|  | Learned a lot from this session |
|  | No comment |
|  | Today’s session was better i felt we actually made progress but would have liked more people to join.. |
|  | I enjoyed the session. I’m not sure if it is totally relevant for me. But I will continue to do it. The reason I was asked to do this was because I had an episode of ex-girlfriends coming back into my life and it basically messed with my head a bit. Since the episode I have felt much better in my mind. Like I said earlier in the session the daytimes I have are usually really good maybe a little bit manic. It’s just when I go to bed and wake up is when I struggle the most. |
| Session 3 | I learnt some new things in the session |
|  | Productive session |
|  | I learned more about reading faces :) |
|  | Spot on |
|  | Good |
|  | Very good content |
|  | No comment |
|  | Slightly longer session this time, which was better. Interaction encouragement was higher and I think sharing the talking between Nicole and Farah was much, much better. |
|  | Good session. Look forward to next week. |
| Session 4 | Productive session |
|  | Good session |
|  | Good |
|  | No comment |
| Session 5 | It was very interesting |
|  | Productive session |
|  | Learned more about jumping CONCLUSIONS and guessing :) |
|  | Good |
|  | No comment |
| Session 6 | Top banana |
|  | Good |
|  | Very interesting |
|  | No comment |
| Session 7 | Productive session again |
|  | Not too sure just didn’t feel as involved as other sessions |
|  | Good |
|  | Very interesting, I liked the role play |
|  | No comment |
| Session 8 | Good conclusion to all sessions |
|  | No comment |

Supplementary figure 1: Results of the mean question scores (with standard error bars) on the presence questionnaire (Witmer et al., 1998) by domain.


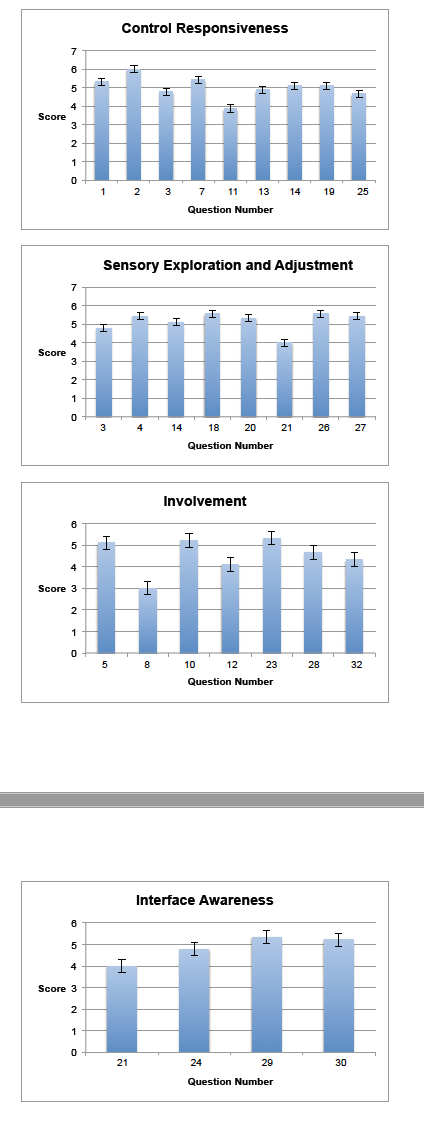

Supplement: Supplementary file 1 [file DataSheet_1.docx]
